# Reviewed article: Chaves ACKA, Meucci RD. Monitoring influenza vaccination coverage among older adults: a rural cohort study, Rio Grande, 2017-2022. Epidemiol Serv Saude. 2026:35;e20250561.

**DOI:** 10.1590/S2237-96222026v35e20250561.a

**Published:** 2026-03-23

**Authors:** Gabriela Gonçalves Amaral

**Affiliations:** 1 Universidade de Pernambuco, , Recife, PE, Brazil

## Peer review A

## Questionnaire

Does the manuscript contain new and significant information to justify publication? Yes

Does the Abstract (Summary) clearly and accurately describe the content of the article? No

Is the problem significant and concisely stated? Yes

Are the methods described comprehensively? No

Are the interpretations and conclusions justified by the results? No

Is adequate reference made to other work in the field? Yes

## Manuscript Structure

Length of article is: Too short

Number of tables is: Adequate

Number of figures is: Adequate

Please state any conflict(s) of interest that you have in relation to the review of this paper. None

Interest: Good

Quality: Average

Originality: Average

Overall: Average

Recommendation: Major Revision

## Comments

Prezados autores,

Espero que estejam bem

Gostaria de parabenizá-los pelo manuscrito, que aborda uma temática relevante para a saúde pública e coletiva, especialmente em um cenário atual marcado pelo aumento da hesitação vacinal e, consequentemente, baixas coberturas vacinais. Além disso, o estudo foca em um público específico de grande importância para o Programa Nacional de Imunizações brasileiro. Espero que as considerações apresentadas contribuam para o aprimoramento do trabalho e para o fortalecimento de sua contribuição científica.

Considerações:

1. TÍTULO:

• Sugestão: Monitoramento da cobertura vacinal contra influenza em idosos da coorte EpiRural: Rio Grande do Sul, 2017 a 2022

2. RESUMO:

• Necessária revisão e adequação, de modo a refletir com fidelidade as modificações sugeridas ao longo do manuscrito. O objetivo apresentado está descrito diferente do apresentado na introdução.

3. PALAVRAS-CHAVES:

• Verifique a pertinência de incluir palavras-chaves existentes no DeCS e que melhor descrevem sua pesquisa, como: cobertura vacinal; Vacinas contra Influenza; idoso; zona rural; Saúde da População Rural; população rural. Os termos: cobertura; influenza; idosos (plural) e rural. não são DeCs.

4. INTRODUÇÃO:

• A seção apresenta considerações relevantes e alinhadas ao objeto do estudo. a) sugiro a substituição da referência 9 pela 6ª edição revisada do Guia de Vigilância em Saúde: Volume 1 (p. 24), que aborda a meta de cobertura vacinal contra a influenza. A proposta se justifica pela atualização e maior fidedignidade teórica da fonte utilizada.

Disponível em: https://www.gov.br/saude/pt-br/centrais-de-conteudo/publicacoes/svsa/vigilancia/guia-de-vigilancia-em-saude-volume-1-6a-edicao/view

• O manuscrito apresenta o seguinte objetivo “estimar a cobertura vacinal contra influenza em idosos da coorte EpiRural de Rio Grande no Rio Grande do Sul, a partir de dados da linha de base em 2017 e em duas ondas de acompanhamento em 2018/19 e 2020/22”. Sugiro a reescrita do objetivo para garantir maior clareza e exatidão quanto ao que foi, de fato, realizado no estudo, ou seja, a estimativa da cobertura vacinal em três momentos distintos da coorte. Uma sugestão seria: “estimar a cobertura vacinal contra influenza em idosos da coorte EpiRural de Rio Grande, com base nos tempos 2017, 2018/2019 e 2020/2022.” Adicionalmente, proponho uma reflexão sobre a necessidade de incluir o “cenário” no enunciado do objetivo. Considerando que se trata de uma coorte identificada nominalmente, o nome da coorte já contempla esse aspecto, sendo mais adequado apresentar o contexto no item específico dos métodos.

5. MÉTODOS:

• Sugiro a leitura e inserção das diretrizes metodológicas propostas para relatos de estudos observacionais em epidemiologia “Strengthening the Reporting of Observational Studies in Epidemiology (STROBE)”, a fim de garantir maior fluidez e robustez na redação da seção de métodos.

• Delineamento do estudo: O manuscrito trata de um recorte de um estudo mais amplo, intitulado “EpiRural Rio Grande: coorte de idosos da área rural de Rio Grande, no Rio Grande do Sul”, cujo objetivo geral é descrever e monitorar os padrões de morbimortalidade e a utilização de serviços de saúde por idosos residentes na zona rural do município. Por ser um recorte, os autores classificam o presente estudo também como uma coorte prospectiva. Para que o recorte em questão se configurasse como um estudo de coorte propriamente dito, seria necessário, por exemplo, acompanhar os “novos registros” de aplicação da vacina entre os idosos que não haviam se vacinado em anos anteriores, a fim de estimar a incidência do desfecho “vacinação contra influenza”. No entanto, essa abordagem se mostra metodologicamente complexa (não impossível), considerando que se trata de uma vacina recomendada anualmente. Em continuação, o desfecho analisado neste trabalho “cobertura vacinal contra influenza” foi estimado de maneira transversal, por meio da prevalência autorreferida da vacinação nos três momentos avaliados. Assim, caracteriza-se como um estudo transversal analítico, derivado de uma coorte, com o objetivo de analisar a cobertura vacinal ao longo do tempo, descrevendo sua evolução entre os anos de 2017, 2018/2019 e 2020/2022. Sugiro a análise pelos autores e possível revisão para garantir que o delineamento informado condiz com a pesquisa.

• Contexto: Recomendo a inserção de maiores informações sobre o contexto do estudo. Sugiro incluir brevemente dados que dimensionem o cenário investigado, como a área territorial considerada “urbana” e “rural no município, e outras informações relevantes que permitam compreender a dimensão do estudo e oferecer subsídios para comparações com outros contextos semelhantes.

• Participantes: Sugiro incluir a justificativa para o recorte da faixa etária (pessoas com 60 anos ou mais). Qual referência foi utilizada para essa definição? Foi o Estatuto do Idoso, o Ministério da Saúde, a Organização Mundial da Saúde, ou outra instituição)?

Além disso, é importante abordar na introdução uma breve explicação sobre quem são considerados idosos no Brasil, considerando que leitores/órgãos públicos de outros países podem adotar critérios diferentes para classificar uma pessoa como “idosa”.

• Tamanho do estudo: Sugiro a realocação do item “tamanho do estudo” para após de “contexto”.

• Tamanho do estudo: O tamanho amostral referido diz respeito apenas ao terceiro momento da coorte (2020/2022)? A amostra apresentada refere-se apenas ao tempo final da coorte (651 idoso). No entanto, o estudo propõe analisar a cobertura vacinal nos três momentos da coleta, o que pressupõe diferentes tamanhos amostrais em função das perdas ao longo do seguimento. Como isso foi conduzido metodologicamente? Analisou-se apenas os 651 participantes nos três tempos? Caso sim, sugiro explicitar esse critério. O desfecho pode ter ocorrido também entre os idosos nos tempos 1 e 2, mesmo entre aqueles que não necessariamente permaneceram na coorte até o tempo final. Caso não seja essa a abordagem adotada, sugiro revisar a redação para tornar mais claro o plano de amostragem e os critérios utilizados para definição do tamanho amostral.

• Métodos estatísticos: Na passagem “Foram mantidas no modelo final variáveis com p-valor < 0,20 para controle de potenciais fatores de confusão”, sugiro revisar a redação, pois parece haver uma imprecisão. Não seria o caso de indicar que variáveis com p-valor < 0,20 na análise bivariada foram levadas para o modelo multivariado, e que, no modelo final, foram mantidas aquelas com p-valor < 0,05.

• Métodos estatísticos: O desfecho utilizado na análise multivariada foi a apresentação das três doses da vacina? Essa informação não está clara nem descrita na seção de métodos, nos resultados ou nas tabelas.

• Métodos estatísticos: Sugiro também uma revisão da redação, de modo a tornar mais clara a análise estatística implementada, bem como a ordem dos testes utilizados.

6. RESULTADOS:

• Confirma-se no primeiro parágrafo que “foram incluídos 651 idosos que participaram da linha de base e das duas ondas de acompanhamento...”, o que indica que os demais idosos que não participaram das três etapas da coorte foram excluídos da análise. No entanto, esse critério de exclusão não está claramente explicitado na seção de métodos. Essa escolha pode introduzir um viés, considerando que esses indivíduos poderiam, pontualmente, ter se vacinado em algum dos momentos avaliados, o que impactaria nos dados de cobertura vacinal. A menos que o objetivo do estudo seja analisar exclusivamente os idosos que permaneceram em todas as fases da coorte, a exclusão dos que saíram ao longo do tempo precisa ser justificada de forma mais clara. Sugiro uma reavaliação e eventual reescrita.

• A medida de associação apresentada “prevalência” como no trecho “a prevalência autorreferida de vacinação contra influenza nos últimos 12 meses aumentou ao longo do tempo: 71,5% na linha de base, 80,4% no primeiro acompanhamento e 85,7% no último acompanhamento, realizado durante a pandemia de Covid-19 (Gráfico 1)”, não condiz com o tipo de delineamento utilizado (coorte). Esse uso retoma a necessidade de reconsiderar a sugestão de alteração do delineamento para estudo transversal analítico, uma vez que, na prática, não estão sendo exploradas medidas de incidência ou de associação próprias de estudos longitudinais.

7. DISCUSSÃO:

• No trecho “Identificou-se um aumento progressivo na prevalência autorrelatada de vacinação contra influenza entre idosos residentes em áreas rurais ao longo do período analisado”, embora tenha sido observado esse aumento, a cobertura vacinal ainda permanece abaixo da meta estipulada pelo PNI. Essa informação é relevante e não foi discutida no texto.

• Realocar o parágrafo de “limitações para o final da discussão, antes da conclusão.

• No trecho “os resultados deste estudo contrastam com a tendência observada no Brasil, onde as coberturas vacinais em idosos caíram progressivamente após 2020, ficando abaixo da meta de 90% estabelecida pelo Ministério da Saúde (9)”. Não seria mais adequado o uso de “corrobora”, já que também foi observado no presente estudo o não alcance da meta de cobertura vacinal estipulada? Ademais, recomendo a inclusão de uma ou mais referências que sustentem a afirmação da “tendência” relatada.

• No trecho “Os fatores associados à vacinação identificados neste estudo como não trabalhar, não fumar, ter comorbidades, fazer uso de medicamentos contínuos e buscar serviços de saúde, estão em consonância com achados de estudos anteriores em diferentes contextos (Brasil, Canadá, Austrália, Tailândia, Polônia, Sérvia, Hungria, Espanha, México)”. Sugiro inserir as referências na localização correspondente a cada cenário apresentado, em vez de agrupá-las apenas ao final como uma compilação. Isso facilita a compreensão e confere maior precisão à vinculação entre os dados e suas fontes.

• De modo geral sugiro um maior aprofundamento na discussão dos resultados, fortalecendo a articulação entre os dados apresentados e a literatura científica. Os fatores associados encontrados foram discutidos de forma bastante compacta, reunidos em um único parágrafo, o que compromete a construção de uma análise crítica e posicionada por parte dos autores. A discussão ocorreu de maneira generalista, com comparações pontuais com outros cenários e sugestões de estratégias para ampliação da adesão vacinal, sem aprofundamento sobre a relevância, implicações ou possíveis mecanismos explicativos para os achados. Sugiro expandir e qualificar essa discussão, de modo a evidenciar o diálogo entre os resultados e a literatura, bem como a reflexão crítica dos autores.

8. TABELAS:

• Sugiro não utilizar abreviações nos nomes das variáveis.

